# Pemafibrate Ameliorates Liver Dysfunction and Fatty Liver in Patients with Non-Alcoholic Fatty Liver Disease with Hypertriglyceridemia: A Retrospective Study with the Outcome after a Mid-Term Follow-Up

**DOI:** 10.3390/diagnostics11122316

**Published:** 2021-12-09

**Authors:** Suguru Ikeda, Takaaki Sugihara, Takuya Kihara, Yukako Matsuki, Takakazu Nagahara, Tomoaki Takata, Sonoko Kitao, Tsuyoshi Okura, Kazuhiro Yamamoto, Hajime Isomoto

**Affiliations:** 1 Department of Gastroenterology and Nephrology, Faculty of Medicine, Tottori University, Yonago 683-8504, Japan; ikeda_suguru@tottori-u.ac.jp (S.I.); t.kihara@tottori-u.ac.jp (T.K.); matsukiy@tottori-u.ac.jp (Y.M.); t.nagahara@tottori-u.ac.jp (T.N.); t-takata@tottori-u.ac.jp (T.T.); isomoto@tottori-u.ac.jp (H.I.); 2 Department of Cardiovascular Medicine and Endocrinology and Metabolism, Faculty of Medicine, Tottori University, Yonago 683-8504, Japan; kitao4924@tottori-u.ac.jp (S.K.); ohkura@tottori-u.ac.jp (T.O.); ykazuhiro@tottori-u.ac.jp (K.Y.)

**Keywords:** non-alcoholic fatty liver disease, non-alcoholic steatohepatitis, hypertriglyceridemia, pemafibrate, liver function, liver fibrosis

## Abstract

Non-alcoholic fatty liver disease (NAFLD) is a chronic liver disease related to metabolic syndrome. No standard pharmacological treatment has yet been established. We retrospectively evaluated the efficacy of pemafibrate in 16 NAFLD patients (11 men and 5 women; median age, 59 years; range, 27–81 years) who had taken pemafibrate for at least one year. They were all diagnosed with fatty liver according to imaging and clinical criteria. They were administered pemafibrate from October 2018 to October 2021 (median, 94 weeks; range, 56–157 weeks). Serum triglyceride was significantly decreased by −41.9% (342.3 ± 54.0 to 198.9 ± 20.4 mg/dL, *p* < 0.001). Aspartate aminotransferase (AST), alanine aminotransferase, and gamma-glutamyl transferase levels significantly decreased by −42.1% (49.6 ± 7.0 to 28.7 ± 3.4 U/L, *p* < 0.001), −57.1% (65.1 ± 10.8 to 27.9 ± 3.7 U/L, *p* < 0.001), and −43.2% (68.9 ± 10.9 to 39.1 ± 5.3 U/L, *p* < 0.05), respectively. The AST to platelet ratio (APRI) (0.8 ± 0.1 to 0.4 ± 0.1, *p* < 0.001) and fibrosis based on four factors (FIB-4) index (1.8 ± 0.3 to 1.4 ± 0.2, *p* < 0.05) also significantly decreased. Liver attenuation (39.1 ± 1.2 to 57.8 ± 2.7 HU, *p* = 0.028) and liver/spleen ratio (0.76 ± 0.04 to 1.18 ± 0.02, *p* = 0.012) significantly improved in three patients, as assessed by computed tomography. In conclusion, pemafibrate significantly improves serum triglyceride levels, liver function, FIB-4 index, APRI, and fatty liver in NAFLD patients with hypertriglyceridemia.

## 1. Introduction

Non-alcoholic fatty liver disease (NAFLD)/steatohepatitis (NASH) has recently emerged as a common public health problem [1]. The prevalence of NAFLD is approximately 25% of the global population, and it is increasing in the Asia Pacific region [2]. Guidelines for the management of NAFLD were proposed by the European Association for the Study of the Liver in 2016 and the American Association for the Study of Liver Diseases in 2018 [3,4]. The Japanese Society of Gastroenterology and the Japan Society of Hepatology have recently launched the newest guidelines for NAFLD/NASH management [5]. However, there are no recommendations regarding pharmacological treatment for NAFLD/NASH.

Peroxisome proliferator-activated receptors (PPARs) form a subfamily of the nuclear receptor superfamily [6]. There are three different types of PPAR (α, δ, and γ). In particular, in NAFLD patients, increased expression of PPARγ is observed, which leads to augmented hepatic triglyceride storage and de novo lipogenesis [7]. On the other hand, PPARα was initially identified as the molecular target of xenobiotics inducing peroxisome proliferation in rodents [8]. It has been well-established as a critical modulator of lipid transport and metabolism, notably mitochondrial and peroxisomal fatty acid beta-oxidation [9,10]. Activation of PPARα promotes the uptake, utilization, and catabolism of fatty acids [11], which can potentially be beneficial in NAFLD patients. PPARα modulation has been considered a key treatment strategy for metabolic diseases, including NAFLD [12].

Pemafibrate (K-877, Parmodia^®^ tablet, Kowa Company, Ltd., Nagoya, Japan), a selective PPARα modulator (SPPARMα), was approved for the treatment of hyperlipidemia in July 2017 and released in June 2018 in Japan. The difference between previously released fibrates and pemafibrate is the high selectivity of the latter toward PPARα [13]. PPARα activation by pemafibrate was reported to be >2500 times stronger than that by fenofibric acid because of its Y-shaped structure, unlike conventional fibrates. This high selectivity enables pemafibrate to be used at a reduced dosage.

We have previously reported that the short-term (median six months) administration of pemafibrate dramatically ameliorates liver dysfunction, assessed based on liver function tests (LFTs) in patients with NAFLD with hypertriglyceridemia [14]. This was the first report of the efficacy of pemafibrate on LFTs in NAFLD. Subsequently, other studies also demonstrated that pemafibrate can improve liver function in NAFLD [15,16,17,18,19]. However, its efficacy on fatty liver has not been elucidated.

In this study, we aimed to retrospectively evaluate the efficacy of pemafibrate on LFTs and fatty liver findings in patients with NAFLD with hypertriglyceridemia.

## 2. Materials and Methods

### 2.1. Study Design and Protocols

**Figure 1:**
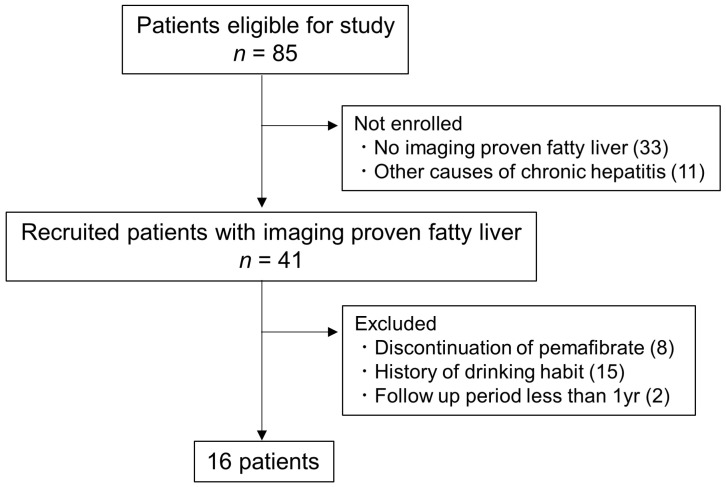
Flowchart of patient selection. Initially, there were 85 eligible patients. Thirty three patients were not enrolled because they had not been proven to have fatty liver through imaging. Patients with other causes of chronic hepatitis were also not enrolled. Patients who stopped pemafibrate for any reason and/or with a history of drinking and short duration of using pemafibrate were excluded from the study. Finally, 16 patients were selected for this study.

This was a single-center, retrospective, observational study enrolling a total of 85 patients in our hospital who were administered pemafibrate between October 2018 and October 2021. Then, only patients with fatty liver diagnosed by imaging (ultrasonography, US, or computed tomography, CT) were selected for the study. The definition of fatty liver by imaging was as described in our previous report [14]. Patients who had other causes of chronic hepatitis (hepatitis B virus, hepatitis C virus, autoimmune hepatitis, primary biliary cholangitis) were not enrolled. Patients who discontinued pemafibrate for any reason, had a history of drinking (ethanol consumption of >20 g/day for women and >30 g/day for men), or took pemafibrate for a short duration (<1 year) were excluded from the study. Finally, we selected 16 patients (11 men and 5 women) (Figure 1). Fourteen patients were overlapped in this study and our previous study [14]. We obtained each patient’s information concerning gender, age, height, and body weight and then calculated their body mass index (BMI), both pre-treatment and most recent. We also collected information about concomitant medications that are considered useful for NAFLD/NASH treatment as follows: dipeptidyl peptidase-4 (DPP4) inhibitor, metformin, sodium-glucose cotransporter 2 (SGLT2) inhibitor, ursodeoxycholic acid (UDCA), eicosapentaenoic acid (EPA), statin, and ezetimibe. Fasting laboratory data including triglyceride (TG), high-density lipoprotein (HDL) cholesterol, low-density lipoprotein (LDL) cholesterol, aspartate aminotransferase (AST), alanine aminotransferase (ALT), and gamma-glutamyl transferase (GGT), fibrosis based on four factors (FIB-4) index (age, AST, ALT, and platelet values [20,21,22]), and AST to platelet ratio index (APRI) [23,24] were also collected. Patients who had an abdominal CT scan twice, at least one year apart, were selected and compared for liver attenuation (Hounsfield unit, HU) and the liver/spleen (L/S) ratio. The HU of the liver and spleen was measured using regions of interest (ROIs) greater than 100 mm^2^ in the area, according to a previous report [25]. Three ROIs were placed in the right liver lobe anteroposteriorly and one ROI in the spleen. The L/S ratio was calculated by taking the mean liver HU and dividing it by the spleen HU value.

### 2.2. Statistical Analysis

Skewness–kurtosis was used to verify the data distribution. The paired *t*-test or the Wilcoxon signed-rank test was applied for comparing two paired groups depending on the data distribution. Welch’s *t*-test was applied for comparing two independent groups. All statistical tests were performed using StatFlex (Windows ver. 6.0; Artech, Osaka, Japan). Values are expressed as median (range) or mean with standard error of the mean (SEM). Statistical significance was set at *p* < 0.05.

## 3. Results

### 3.1. Baseline Characteristics of the Patients

The median age of the patients was 59 years (range, 27–81 years). Nine patients had type 2 diabetes (T2DM) (56.3%). Ten patients (62.5%) had been diagnosed with fatty liver by US, and six patients (37.5%) had been diagnosed with fatty liver by CT. Four patients had been diagnosed with NASH by liver biopsy. Pre-pemafibrate treatment laboratory values were as follows: TG 342.3 ± 54.0 mg/dL, HDL-cholesterol 47.3 ± 2.4 mg/dL, LDL-cholesterol 113.5 ± 10.0 mg/dL, AST 49.6 ± 7.0 U/L, ALT 65.1 ± 10.8 U/L, and GGT 68.9 ± 10.9 U/L. DPP4 antagonist, metformin, SGLT2 inhibitor, EPA, statin, ezetimibe, and UDCA had been already prescribed in five (31.3%), three (18.8%), six (37.5%), four (25.0%), six (37.5%), seven (43.8%), and four (25%) patients, respectively. Pemafibrate was administered in 1 patient (6.3%) at 0.1 mg qd (once daily), 13 (81.3%) at 0.1 mg bid (twice daily), and 2 (12.5%) at 0.2 mg bid. The median duration of administration was 94 weeks (range, 56–157 weeks) (Table 1).

### 3.2. Changes in LFTs and Fibrosis Markers

**Figure 2:**
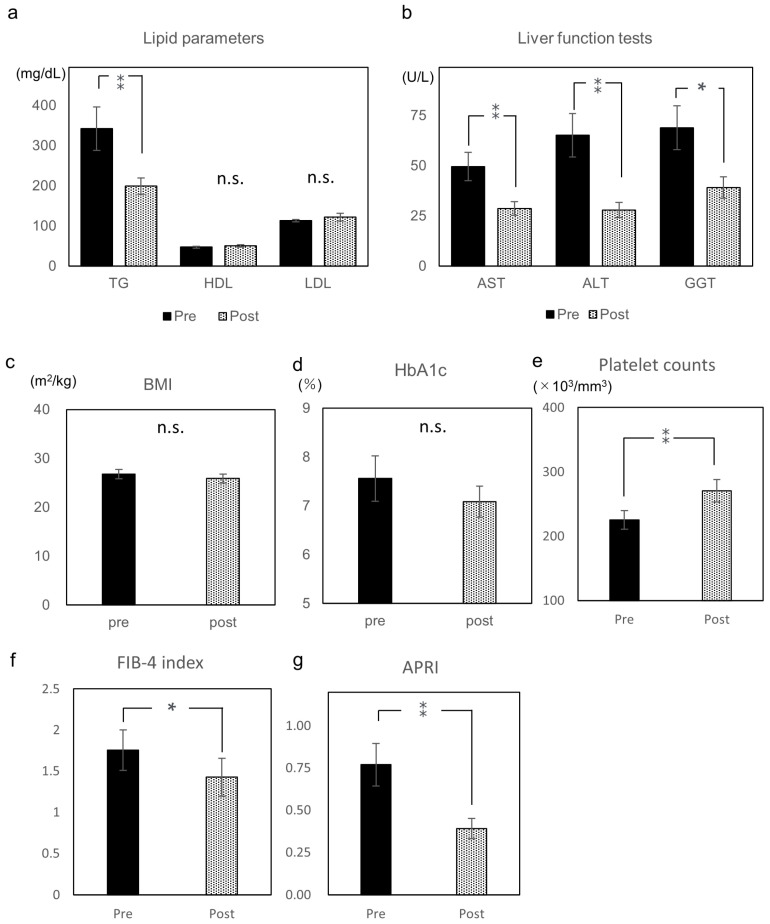
Pre and post laboratory and physical data of pemafibrate treatment for median 94 weeks. (**a**) Triglyceride, high-density lipoprotein cholesterol, and low-density lipoprotein cholesterol. (**b**) Aspartate aminotransferase, alanine aminotransferase, and gamma-glutamyl transferase. (**c**) Body mass index (BMI). (**d**) Glycated hemoglobin (HbA1c). (**e**) Platelet counts. (**f**) Fibrosis based on four factors (FIB-4) index. (**g**) Aspartate aminotransferase to platelet ratio index (APRI). Data are expressed as mean with standard error of the mean (SEM). n.s. not significant, * *p* < 0.05, ** *p* < 0.01.

Serum TG significantly decreased after pemafibrate treatment by −41.9% (342.3 ± 54.0 to 198.9 ± 20.4 mg/dL, *p* < 0.001); however, serum HDL-cholesterol and LDL-cholesterol levels did not significantly differ (HDL, 47.3 ± 2.4 to 50.3 ± 2.8 mg/dL; LDL, 113.5 ± 10.0 to 121.6 ± 9.5 mg/dL) (Figure 2a). Serum AST, ALT, and GGT levels decreased after pemafibrate treatment by −42.1% (49.6 ± 7.0 to 28.7 ± 3.4 U/L, *p* < 0.001), −57.1% (65.1 ± 10.8 to 27.9 ± 3.7 U/L, *p* < 0.001), and −43.2% (68.9 ± 10.9 to 39.1 ± 5.3 U/L, *p* < 0.05), respectively (Figure 2b). BMI and HbA1c did not significantly differ (Figure 2c,d). Platelet counts significantly increased (225.3 ± 14.3 to 270.6 ± 17.6 × 10^3^/mm^3^, *p* < 0.001) (Figure 2e). FIB-4 index and APRI significantly decreased (FIB-4 index, 1.8 ± 0.3 to 1.4 ± 0.2, *p* < 0.05; APRI, 0.8 ± 0.1 to 0.4 ± 0.1, *p* < 0.001) (Figure 2f,g).

### 3.3. Changes in Liver Attenuation and L/S Ratio by CT Imaging

Among the 16 patients, three patients (Case #12, #56, and #72) who underwent an abdominal CT scan twice within at least a one-year interval were selected (Table 2).

**Figure 3:**
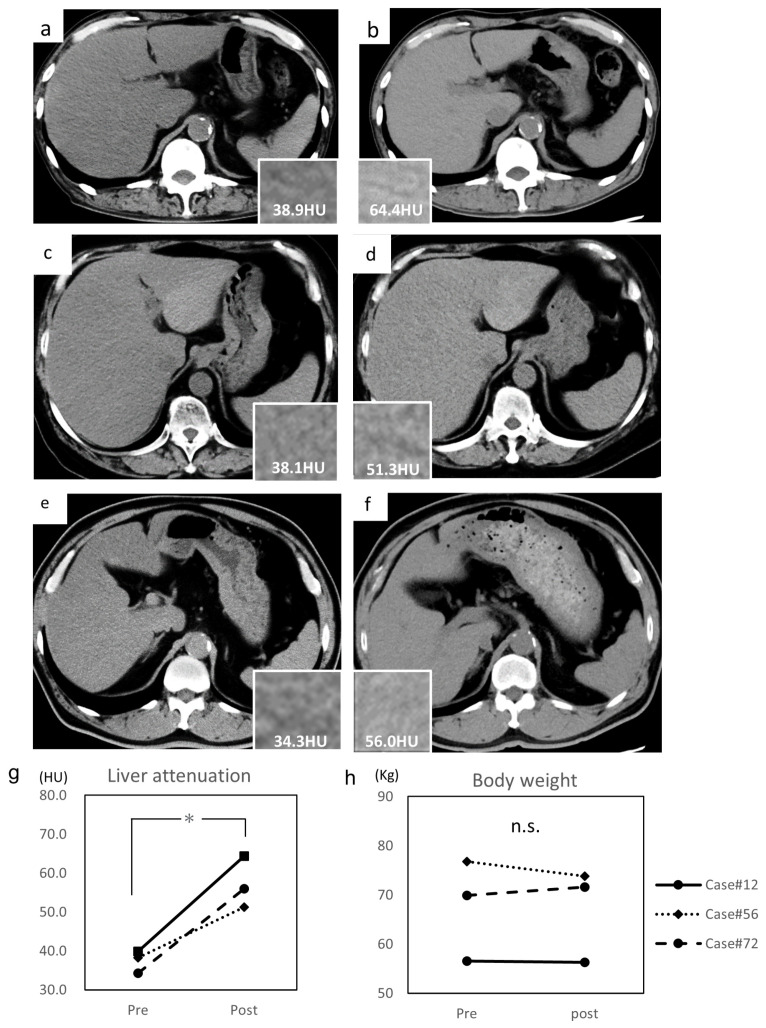
Alteration of liver attenuation assessed by CT imaging. (**a**) Pre-treatment abdominal CT of Case #12, (**b**) post-treatment abdominal CT of Case #12, (**c**) pre-treatment abdominal CT of Case #56, (**d**) post-treatment abdominal CT of Case #56, (**e**) pre-treatment abdominal CT of Case #72, and (**f**) post-treatment abdominal CT of Case #72. (**g**) Alteration of liver attenuation assessed by CT imaging before and after treatment. Liver attenuations are indicated in each column. (**h**) Alteration of body weight before and after treatment. Hounsfield unit, HU. Data are expressed as mean with standard error of the mean (SEM). n.s. not significant, * *p* < 0.05.

The median interval was 95.1 (range, 67–118) weeks. The average pre-treatment liver attenuations of Case #12, #56, and #72 were 38.9 HU (Figure 3a), 38.1 HU (Figure 3c), and 34.3 HU (Figure 3e), respectively. The average post-treatment liver attenuations of Case #12, #56, and #72 were 64.4 HU (Figure 3b), 51.3 HU (Figure 3d), and 56.0 HU (Figure 3f), respectively. The average liver attenuation significantly increased after treatment (39.1 ± 1.2 to 57.8 ± 2.7 HU, *p* = 0.028) (Figure 3g). The L/S ratio also significantly improved (0.76 ± 0.04 to 1.18 ± 0.02, *p* = 0.012). Their TG levels decreased and LFT performance increased (AST, 30.3 ± 4.0 to 20.3 ± 3.1 U/L; ALT, 39.3 ± 4.1 to 20.0 ± 8.2 U/L; GGT, 43.3 ± 4.3 to 27.7 ± 5.2 U/L); however, LDL-cholesterol (135.3 ± 22.4 to 137.7 ± 26.7 mg/dL), HbA1c (8.1 ± 0.7 to 7.7 ± 0.5%), and body weight (67.7 ± 4.9 to 67.2 ± 4.5 kg) (Figure 3h) did not significantly differ.

## 4. Discussion

In this study, we demonstrated that pemafibrate significantly decreases TG levels and ameliorates liver dysfunction in NAFLD with hypertriglyceridemia for a median of 94 weeks of administration. Surprisingly, even fatty liver significantly improved in some patients. To our knowledge, this is the first report demonstrating the possible effects of pemafibrate on fatty liver in patients with NAFLD.

After our first study, which demonstrated that pemafibrate ameliorated liver dysfunction in patients with NAFLD with hypertriglyceridemia [14], five studies demonstrated the efficacy of pemafibrate on LFTs, consistent with our results [15,16,17,18,19]. Two of them were prospective [15,19], and the other four, including ours, were retrospective studies [14,16,17,18]. The most extended study spanned 72 weeks [19].

Seko et al. [15] conducted a single-arm prospective study in 20 NAFLD patients for 12 weeks and demonstrated that pemafibrate (0.1 mg) twice a day significantly decreases ALT, GGT, and TG levels and increases the HDL-cholesterol level. They indicated that BMI and insulin resistance are not correlated with changes in ALT levels. Shinozaki et al. [16] conducted a retrospective observational study in 38 patients with NAFLD for three months and demonstrated that pemafibrate (0.1 mg) twice a day significantly decreases the levels of ALT, GGT, and TG and the NAFLD fibrosis score [26] and increases the HDL-cholesterol level. Hatanaka et al. [17,18] conducted two retrospective studies. The first study [17] was in 10 biopsy-proven NASH patients administered pemafibrate (0.1 mg) twice a day for 24 weeks; their AST and ALT values were significantly decreased, especially in NASH patients with significant activity and advanced fibrosis. The second study [18] was in 31 US-proven NAFLD patients for 48 weeks, including nine NASH patients from the previous study; they demonstrated that pemafibrate improves the FAST score [27] based on the AST, LSM, and CAP levels. One recent randomized study in 118 MRI-proven NAFLD patients for 72 weeks [19] showed that pemafibrate treatment could ameliorate ALT, GGT, and ALP levels and liver stiffness. This RCT did not show statistical differences in liver fat content; however, the fat content decreased in the pemafibrate group (placebo +0.2% vs. pemafibrate −5.0%) in 72 weeks. The reported efficacy of pemafibrate on hepatic steatosis is not consistent [28,29]. However, pemafibrate can theoretically reduce the fat content by activating PPARα. Our result implies that pemafibrate can also reduce fat content after longer-term administration regardless of weight loss or diabetes status. Further investigation is required in this direction.

In the present study, the mean values of APRI and FIB-4 index decreased. Our previous study for six months could not demonstrate the decrease in FIB-4 index; however, the more extended treatment duration, in the present study, decreased both parameters. Both formulas include platelet counts. As observed in our previous study for six months, pemafibrate treatment significantly increased platelet counts. The prospective study by Seko et al. [15] also demonstrated significantly increased platelet counts. Platelets play an important role not only in hemostasis but also in inflammatory reactions, angiogenesis, wound healing, and the resolution of inflammation [30]. Platelets are reported to be active participants in the process of liver inflammation and play a central role in the progression from simple steatosis to NASH [31,32]. Considering other hepatic parameters, increased platelet counts are considered the result of the resolution of inflammation in the liver. This partially explains the significant reduction in APRI and FIB-4 index. Other studies also agreed with the efficacy of pemafibrate in hepatic fibrosis.

Thiazolidinediones, SGLT2 inhibitors, metformin, and DPP4 antagonists have also been reported to have favorable effects on NAFLD in T2DM patients [33,34]. In our study, there were five, three, and four patients who had already taken DPP4 antagonists, metformin, and SGLT2 inhibitors at the beginning of pemafibrate treatment, respectively. Two patients were given SGLT2 inhibitors after the administration of pemafibrate (from the 7th and 11th months). DPP4 antagonists, metformin, and SGLT-2 inhibitors were stopped in two, one, and one patient during the treatment, respectively. No patient had been prescribed thiazolidinediones. There were no significant differences in the LFTs considering DPP4 antagonists and SGLT2 inhibitors. The patients who had already been treated with DDP4 antagonists and SGLT2 inhibitors before starting pemafibrate may not have shown differences in LFTs because their diabetic status had already improved. Therefore, we also compared the pre- and post-HbA1c between with and without DPP4 antagonists or SGLT2 inhibitors; however, there were no differences in pre-HbA1c (with vs. without DPP4 antagonists: 8.0 ± 0.5 vs. 7.8 ± 0.9%, *p* = 0.8129; with vs. without SGLT-2 inhibitors: 7.2 ± 0.6 vs. 8.0 ± 0.6%, *p* = 0.4790) and post-HbA1c (with vs. without DPP4 antagonists: 7.5 ± 0.4 vs. 7.1 ± 0.4%, *p* = 0.5241; with vs. without SGLT2 inhibitors: 7.3 ± 0.6 vs. 7.3 ± 0.4%, *p* = 0.9659). In three patients who had metformin, only the GGT levels significantly decreased compared with those who did not (43.1 ± 5.9 vs. 22 ± 3.9, *p* = 0.03). However, the GGT levels were relatively lower in the metformin group from the beginning (74.1 ± 13.0 vs. 46.3 ± 4.3, *p* = 0.08). Therefore, the significantly decreased GGT levels in the metformin group can also be explained by pemafibrate’s efficacy.

Among drugs for dyslipidemia, several studies have demonstrated the efficacy of β-Hydroxy β-methylglutaryl-CoA reductase inhibitors (statins) on NAFLD/NASH patients [35,36,37,38]. Statins are recommended for NAFLD/NASH patients with hypercholesterolemia in the new guidelines of Japan [5]. Statins can ameliorate LFTs; however, consistent histological improvements are still controversial. Pemafibrate is the first fibrate that can be used safely in combination with a statin. In this study, six patients had been prescribed a statin. However, there were no significant differences in the LFTs considering drugs for dyslipidemia, including statins. UDCA is not recommended in the guidelines [5]. Four patients had taken UDCA; however, there were no significant differences in the LFTs considering UDCA administration.

All three patients with follow-up CT data had T2DM and hypercholesteremia and had already been treated with antidiabetic drugs or statins at the beginning of pemafibrate treatment. In Case #52, metformin was started at the same time as pemafibrate. However, a systematic review indicated that metformin yielded no difference from a placebo in steatosis, fibrosis, NAFLD activity score, or the resolution of NASH [34]. After adding pemafibrate, the patients’ TG levels, liver dysfunction, and non-invasive liver fibrosis scores decreased. Only the additional pemafibrate can explain the improvement in liver attenuations and L/S ratios.

Three (17.6%) so-called “lean” NAFLD patients (median BMI 21 kg/m^2^) were included. One (BMI 19.2 kg/m^2^) presented with follow-up CT data (Case #12). NAFLD can develop in the absence of obesity (BMI within the ethnicity-specific cutoff of 25 kg/m^2^ in Caucasians and 23 kg/m^2^ in Asians) [39]. The “lean” phenotype is variable and not completely understood, and treatment recommendations are not provided for “lean” NAFLD patients. The values of serum TG, AST, ALT, and GGT decreased after pemafibrate treatment by −32.9% (248.3 ± 39.3 to 166.7 ± 40.9 mg/dL), −62.2% (54.7 ± 16.2 to 20.7 ± 2.7 U/L), −73.0% (70.3 ± 38.5 to 19.0 ± 7.4 U/L), and −64.0% (77.7 ± 27.0 to 28.0 ± 2.1 U/L), respectively. Two of the patients received only DPP4 antagonists for their T2DM. In this regard, DPP4 antagonists and pemafibrate have the potential to benefit even “lean” NAFLD patients with T2DM and hypertriglyceridemia.

Our study had some limitations. It was a small-sized, retrospective, observational study. Selection bias could not be avoided because this study enrolled only those patients diagnosed with NAFLD based on imaging data. Therefore, it is difficult to draw a solid conclusion in clinical terms. Liver biopsy is undoubtedly the gold standard for diagnosing NASH at present. In this study, only four patients had a biopsy performed. However, liver biopsy has several drawbacks, and its role is becoming limited [5]. Non-invasive tools for assessing fibrosis can guide the decision regarding whether to perform a liver biopsy in patients with NAFLD [40].

This is the most extensive study, and the results of the present study are consistent with previous reports [15,16,17,18,19]. According to the existing evidence, including our study, pemafibrate can undoubtedly ameliorate liver dysfunction assessed by LFTs (especially ALT, GGT, and ALP levels) in NAFLD patients with hypertriglyceridemia after only 12 weeks of administration. Pemafibrate also improves liver fibrosis after more than 48 weeks of treatment. Notably, no remarkable adverse effects were reported in these studies.

## 5. Conclusions

We demonstrated that pemafibrate, the new SPPARMα, decreases liver dysfunction assessed by LFTs in patients with NAFLD/NASH with hypertriglyceridemia and possibly reduces liver fat. Pemafibrate has the potential to be the first standard medication for NAFLD.

## Figures and Tables

**Table 1:** Characteristics of the 16 patients treated with pemafibrate.

	*n* = 16	
Male/Female	11:5	
Age (years)	59 (27–81)	
Body height (m)	1.62 (1.46–1.77)	
Pre-treatment bodyweight (kg)	70.4 (48.6–101.7)	
Pre-treatment BMI (kg/m^2^)	26.8 (19.2–33.8)	
Comorbidities		
T2DM	9	(56.3)
Chronic hepatitis B *	1	(6.3)
CAD	1	(6.3)
IBD	1	(6.3)
Other ^†^	1	(6.3)
Imaging modalities		
US	10	(62.5)
CT	6	(37.5)
Biopsy-proven NASH	4	(25.0)
Pre-treatment laboratory values		
TG (mg/dL)	342.3 ± 54.0	
HDL-cholesterol (mg/dL)	47.3 ± 2.4	
LDL-cholesterol (mg/dL)	113.5 ± 10.0	
AST (U/L)	49.6 ± 7.0	
ALT (U/L)	65.1 ± 10.8	
GGT (U/L)	68.9 ± 10.9	
FIB-4 index	1.8 ± 0.3	
APRI	0.8 ± 0.1	
Concomitant medications		
DPP4 antagonist	5	(31.3)
Metformin	3	(18.8)
SGLT2 inhibitor	6	(37.5)
EPA	4	(25.0)
Statin	6	(37.5)
Ezetimibe	7	(43.8)
UDCA	4	(25.0)
Dosage of pemafibrate per day		
0.1 mg	1	(6.3)
0.2 mg	13	(81.3)
0.4 mg	2	(12.5)
Duration of pemafibrate administration (weeks)	94 (56–157)	

APRI, aspartate aminotransferase to platelet ratio index; AST, aspartate aminotransferase; ALT, alanine aminotransferase; BMI, body mass index; CAD, coronary artery disease; CT, computed tomography; DPP4, dipeptidyl peptidase-4; EPA, eicosapentaenoic acid; GERD, gastroesophageal reflux disease; GGT, gamma-glutamyl transpeptidase; HDL, high-density lipoprotein; IBD, inflammatory bowel disease; LDL, low-density lipoprotein; MRI, magnetic resonance imaging; SGLT2, sodium-glucose cotransporter 2; T2DM, type 2 diabetes mellitus; TG, triglyceride; UDCA, ursodeoxycholic acid; US, ultrasonography. * HBV DNA is controlled under detection by nucleotide analog treatment. ^†^ Ovarian insufficiency. Data are expressed as median (range) or mean ± SEM. Numbers in parentheses refer to the percentage of patients.

**Table 2:** Characteristics of the three cases with CT follow-up.

Male/Female	2:1
Age (years)	63 (59–64)
Pre-treatment BMI (kg/m^2^)	24.8 (19.2–29.8)
Comorbidities	
T2DM	3
Pre-treatment laboratory values	
TG (mg/dL)	265.7 ± 45.7
HDL-cholesterol (mg/dL)	42.3 ± 2.2
LDL-cholesterol (mg/dL)	135.3 ± 22.4
AST (U/L)	30.3 ± 4.0
ALT (U/L)	39.3 ± 4.1
GGT (U/L)	43.3 ± 4.3
FIB-4 index	1.38 ± 0.17
APRI	0.46 ± 0.04
Concomitant medications	
DPP4 antagonist	2
Metformin	1
SGLT2 inhibitor	1
EPA	1
Statin	2
Ezetimibe	2
Dosage of pemafibrate per day	
0.1 mg	1
0.2 mg	1
0.4 mg	1
Duration of pemafibrate administration (weeks)	95.1 (67.0–118.0)

APRI, aspartate aminotransferase to platelet ratio index; AST, aspartate aminotransferase; ALT, alanine aminotransferase; BMI, body mass index; CT, computed tomography; DPP4, dipeptidyl peptidase-4; EPA, eicosapentaenoic acid; GGT, gamma-glutamyl transpeptidase; HDL, high-density lipoprotein; IBD, inflammatory bowel disease; LDL, low-density lipoprotein; SGLT2, sodium-glucose cotransporter 2; T2DM, type 2 diabetes mellitus; TG, triglyceride. Data are expressed as median (range) or mean ± SEM.
